# Reversed Chloroquine Molecules as a Strategy to Overcome Resistance in Malaria

**DOI:** 10.2174/156802612799362968

**Published:** 2012-03

**Authors:** David H Peyton

**Affiliations:** Department of Chemistry, Portland State University, Portland, OR 97207-0751

**Keywords:** Antimalarial, chloroquine, *Plasmodium falciparum*, drug development, drug design, structure activity relationship, malaria, drug resistance.

## Abstract

This short review tells the story of how Reversed Chloroquine drugs (RCQs) were developed. These are hybrid molecules, made by combining the quinoline nucleus from chloroquine (CQ) with moieties which are designed to inhibit efflux *via* known transporters in the membrane of the digestive vacuole of the malaria parasite. The resulting RCQ drugs can have potencies exceeding that of CQ, while at the same time having physical chemical characteristics that may make them favorable as partner drugs in combination therapies. The need for such novel antimalarial drugs will continue for the foreseeable future.

## INTRODUCTION

Chloroquine (CQ) was a truly remarkable drug, and the standard of care for malaria over about half a century, beginning in the late 1940s. Speaking about the discovery of CQ in a Review Lecture published in 1963, G. Robert Coatney stated [1], “Results since 1946 have made it the drug of choice for malaria the world over. It was the only antimalarial used by our Armed Forces and most of the Allied Forces in Korea. It has since been employed by most of the Free World Forces when deployed in malarious areas and is the drug of choice in the world-wide malaria eradication program under the World Health Organization.”

Of course, that worldwide malaria eradication program did not succeed, and upon its abandonment the situation regarding malaria grew worse. The emergence and subsequent spread of drug resistance in malaria had made the (otherwise favored) malaria drug CQ ineffective throughout most of the world [2]. Combination therapies [3, 4] have now become the standard of care for malaria chemotherapy, though currently available combinations lack CQ’s simplicity & low cost. Because malaria will doubtless continue to evolve resistance even to the newest cures [5, 6], there is, and will be an ongoing need for a development pipeline of effective drugs until eradication of the disease might become a reality.

In response to this challenging situation, we embarked upon a hybrid-drug approach to overcoming malaria’s drug resistance. Chemosensitizers, or reversal agents (RAs), are molecules that reverse resistance to a drug. In the context of this review, the RA is generally an efflux mechanism inhibitor. RAs for CQ turn the susceptibility of CQ-resistant strains back toward that of CQ-sensitive strains [7-11], mainly by inhibiting the transporter of CQ from the digestive vacuole (DV) – *P. falciparum* chloroquine-resistance transporter (PfCRT) [12]. We originally hypothesized and showed that CQ-resistance could be reversed by covalently bonding CQ to a RA, producing a single molecule that we term a ‘reversed-CQ’ (RCQ) compound [13]; this is illustrated in Fig. (**1**). An RCQ compound has a significant advantage over a cocktail of CQ and RA because CQ accumulates strongly in the parasite’s DV, so the linked RA entity accumulates to a much higher concentration than an unlinked RA. The drug:RA ratio is, by definition, 1:1. We have demonstrated the feasibility of this approach by making several dozens of these compounds, and shown that the design is robust and flexible [13-15]. We have generated a large number of potent and novel RCQ compounds that are very effective against both CQ-sensitive and CQ-resistant strains at concentrations far lower than CQ or other similar drugs. *In vitro* cytotoxicity results for these drugs are favorable [14, 15]. In fact, with the better RCQ drugs we have not observed any adverse effects in mice when the drugs are administered orally with an amount well over an order of magnitude above the *in vivo* ED_50_ values, and well above the fatal CQ dose.

What follows is a short story about how the RCQ molecules have developed in our laboratory, and a brief description of a few molecule classes that are closely related to the RCQ design. The reader can refer to other publications for reviews covering fundamentals of malaria’s CQ-resistance [16], the discovery and development of resistance reversal drugs [10, 17], hybrid drugs for malaria [18], as well as hybrid drugs generally [19, 20], in addition to a recent review covering the need for new malaria drugs [21].

## THE “TARGET”: PfCRT

A protein localized to the membrane defining the digestive vacuole (DV) of *P. falciparum* was discovered by the Wellems group at NIH to have mutations that are correlated to CQ-resistance [12]. Since the function of this protein was not known, it was designated as the, “*P. falciparum* Chloroquine Resistance Transporter,” or PfCRT. Although there were multiple mutations found within the locus of the *pfcrt* gene, a key mutation for CQ-resistance was K76T. Most importantly, incorporation of this mutation into a CQ-sensitive strain induced the CQ-resistance trait. This, and several quickly following papers, e.g., [22-25] established the importance of this point mutation in *P. falciparum* CQ-resistance, although other mutations and probably other transporters are known to modulate the trait [16, 22, 25-27].

The structure of PfCRT has been predicted to contain 10 transmembrane helices, with the N- and C- termini extending into the parasite’s cytoplasm, away from the DV [12]. The K76T location is near at the proposed DV-end of the N-terminal (first) transmembrane helix, and so could interact electrostatically with drugs like CQ, which exists as the dication when in the acidic DV; the loss of the positively-charged lysine residue could then form the basis for resistance by reducing this unfavorable interaction such that the drug then can bind and be transported. In any event, the thermodynamics involved in the transport process are not simple, as recently reviewed [27].

## THE FIRST RCQ MOLECULE

The fundamental design of a reversed drug molecule is a hybridization of the original drug with a reversal agent (chemosentizer) that can inhibit the resistance mechanism, in this case the export of the drug by an efflux mechanism. For our initial RCQ drug, we chose a reversal agent moiety that was known to produce a buildup of CQ (pK_a_ ~ 8.1 & 10.2 [28]) within the parasite (and so presumably in the DV; pH ~ 5 [28]). Such molecules, including verapamil (VP), are often thought of by their activities as calcium channel blockers. Although VP was the usual reference for a CQ-resistance reversal agent, it is somewhat complex from a chemical perspective. However a simpler choice, imipramine (pK_a_ ~ 9.5 [29]), was also known to be a potent RA [11, 30-32], and is achiral, unlike VP and many other reversal agents known to act against CQ-resistance. The commercial availability of desimipramine led to a simple synthesis for the molecule, **1**, also achiral and first published in 2006 [13]. The structure and synthetic route are shown in Fig. (**2**). This molecule, to our knowledge, was the first reported hybrid drug that intentionally incorporated a chemical-efflux inhibitor coupled to a drug molecule. When tested *in vitro* against both a CQ-sensitive *P. falciparum* strain (D6) and against a CQ-resistant strain (Dd2), using the SYBR Green I-based fluorescence assay [33], it was found to be more potent than CQ against either strain, even the CQ-sensitive strain. However, RCQ molecule **1** has a very high estimated clogP of nearly 9, as well as a clogD of 4.2 at pH 7.4 (Marvin 5.5.0.0, 2011, ChemAxon (http://www.chemaxon.com) was used for this calculation), and so was not expected to be a practical drug itself. Nonetheless, we evaluated the *in vivo* potential of the RCQ drugs in a small mouse trial using *P. chabaudi*, and found that **1** had >99% suppressive activity at 64 mg/kg/day in a standard 4-day suppressive test. It is important, however, to remember that **1** was a prototype molecule, assembled to test the principle without being optimized. We then returned to the principles of the RA pharmacophore to begin the optimization process.

## SECOND-GENERATION RCQ MOLECULES

The structural essentials for the CQ-resistance reversing RA molecules identified by Bhattacharjee *et al.* in 2002 [34] included, “Two aromatic hydrophobic sites and a hydrogen bond acceptor site, preferably at a side chain nitrogen atom. We took this conclusion, based on a modeling / calculation approach based on a training set of 17 compounds, as license to open up the RA structure to modifications in a guided way that would remove the tricyclic nature of the imipramine-based RA end of molecule **1**. This might reduce or eliminate any central neural system (CNS) activity of the resultant drugs. In addition, there were the questions of water-solubility and oral availability of the drug to address.

We began to evaluate the structural requirements for an RCQ drug by examining the limits to which one could practically vary the “linker” between the quinoline and the RA’s side-chain nitrogen, as well as between that nitrogen and the RA aromatic rings [14]. We found little differences between RCQ molecules that had between 2 and 4 carbons in the linker between the quinoline and the RA’s side-chain nitrogen, and significant changes could be made to the RA structures themselves. A few examples are shown in Table **1**. The length of the chain between the nitrogen and the 2 aromatic groups was varied between 2 and 4 carbons (and changed to a piperazinyl ring) with little change in activity, as well as incorporating a carbonyl (but *not* converting the central nitrogen into an amide). The aromatic rings were converted from phenyls to benzyls, with little change in activity. A recent paper addresses the ability of this moiety to act specifically as an inhibitor of mutated PfCRT [35]. Two other points that arose from this structure-activity relationship (SAR) work were that having a proton on the quinoline’s 4-amino was beneficial, and that having two RA groups was not a strong advantage. So there was great breadth in scope found for RCQ design, and considerations such as pharmacology and cost could then be addressed with few constraints. In fact, the latitude for RCQ design appeared to be much greater than for developing a ‘simple’ RA.

This conclusion underscores the point that there are fundamental differences in the requirements for the design of an effective RA drug, as opposed to RCQ drugs. First, the RCQ molecule’s fundamental activity must be antimalarial activity. The functional requirement of a RA molecule is the ability to inhibit efflux of CQ in a malaria strain; the function of a RA moiety in a RCQ drug is to enhance buildup of the drug in the DV (presumably by decreasing efflux of itself). This latter property does not necessarily require that it be able to inhibit efflux of CQ. In fact, it may be that the best RCQ drugs may not interact with an efflux effector such as PfCRT at all: RCQ drugs could bind other transporters in the DV, or they could simply have lower binding to all such transporters. This is because binding is the first step in transport, so that reduced binding could lead to a higher RCQ drug concentration in the DV, even though it would be a less effective RA for CQ export. Therefore, it may be easier to design an optimized RCQ drug RA than it would be to design the corresponding RA moiety.

## AN IMPROVED RCQ MOLECULE

We continued the SAR work, taking advantage of the above-referenced flexibility to find a molecule, **2**, that has great water solubility, has good *in vitro* antimalarial activity against a variety of CQ-resistant and CQ-sensitive *P. falciparum* strains, and is orally curative in mice. Its IC_50_ values against D6, Dd2, and 7G8 were about 1, 2, and 2 nM, as compared to CQ values of 7, 102, and 106 nM. Having a clogP value of about 3.5 (CQ has a value of about 5), **2** was anticipated to have good water solubility, especially as a salt formulation (e.g., chloride or phosphate). Low cytotoxicity values (in our studies, to mouse spleen lymphocytes), and the resulting favorable therapeutic index values were found for many RCQ molecules. For example, the therapeutic index values for CQ and **2** were found to be about 120 and 4000, respectively (ratio of IC_50_ for mouse spleen lymphocytes / *P. falciparum* strain Dd2). *In vivo* work in mice found that **2** could cure mice of a *P. berghei* infection at an equimolar dose to 30 mg/kg/day for CQ. Remarkably, while CQ kills at < 200 mg/kg in mice, we could orally dose **2** to > 400 mg/kg in mice without evident adverse effects for at least 3 days (unpublished).

## MECHANISM(S) OF ACTION

While the full story about the mode(s) of action for CQ is not certain, it has been widely assumed to involve interaction with heme, a byproduct of hemoglobin digestion by the *Plasmodium* parasite. We therefore evaluated the binding of heme by both CQ and **1** using UV-vis spectroscopy. A binding stoichiometry of one drug molecule to one heme μ-oxo dimer was assumed. At pH 5.7, near the DV pH, the measured binding constant (3 × 10^5^ M^-1^) was found to be the same as measured for CQ (3 × 10^5^ M^-1^). The measured binding constant at pH 7 (also 3 × 10^5^ M^-1^) was also found to be comparable to that which we measured for CQ (1 × 10^6^ M^-1^) under the same conditions. The conclusion is that the RA portion of **1** does not appear to affect the CQ-like binding to heme. This conclusion has been supported by a recent study [36]. Compound **2** Fig. (**3**) also proved to be an avid molecule for the heme µ-oxo dimer, and was about 3-fold more efficient at inhibiting β-hematin formation as Compound **1**. This may indicate that the high potencies of the drugs is a combination of both enhanced accumulation and enhanced interaction with heme, both relative to CQ. We also tested the ability to accumulate in *P. falciparum*-parasitized red cells. In this test, compound **1** did, in fact, accumulate to a stronger degree than did CQ, as measured by the drop of drug concentration in the surrounding medium. Light microscopic analysis of surviving parasites in high concentrations of **1** showed a strongly swelled DV, as well as very little apparent hemozoin crystals. CQ under the same conditions showed the same qualitative effects, but to a lesser extent. All this is consistent with an inhibition of hemozoin formation mechanism for the RCQ molecules, in analogy with the accepted mechanism of CQ, except that the RCQ molecule is generally more potent across the range of effects.

We found that the RCQ drugs’ effectiveness is not due to varying the length of the linker between the quinoline and the central nitrogen; this result is important because others have found that such changes alone can overcome CQ-resistance. Thus, we synthesized a compound **3** Fig. (**3**), which was found to have better antimalarial activity than CQ, and in combination with the work cited above, we concluded that the ability of the RCQ molecules to overcome CQ-resistance by the addition of a RA head group to the 4-aminoquinoline ring is, in fact, independent of the chain length between them, at least if the chain length is between 2 and 4 carbons.

The question of whether the RCQ molecules actually inhibit PfCRT’s ability to export CQ in strains which are CQ-resistant may best be evaluated by measurement systems such as implemented by Martin and Kirk [28, 35]. In fact, molecules such as **1** and **2** do inhibit this ability to inhibit CQ transport by PfCRT^76T^ (unpublished data).

## RELATED MOLECULES

October *et al.* used the chemistry of 3,4-Dihydropyrimidin-2(1H)-ones to construct RCQ molecules [37]; Fig. (**4**). Although such molecules lack the protonatable nitrogen of the classical pharmacophore, this structure did exhibit excellent potency against CQ-sensitive and CQ-resistant malaria strains. Another set of molecules, linking astemizole to the CQ quinoline end likewise proved hopeful [38]. Substitution of adamantyl groups for the phenyls in the RA pharmacophore has proved somewhat successful for others [39] in overcoming *P. falciparum* resistance, as well in our laboratory (unpublished). Screening led to related structures, e.g., [40, 41]. The acridine skeleton has been hybridized to CQ to make related RCQ structures [42]. The dibemethin group was appended to quinolines to make related hybrids [43], based on this group’s ability to inhibit PfCRT-mediated efflux of CQ [35], as outlined above. The drug candidate ferroquine [44, 45] may owe much of its potency to the ability to accumulate in the DV and not be exported by mutated PfCRT.

Even some older work, eg., bisquinolines; Fig. (**4**) [46, 47], including piperaquine [48], make up an interesting set of compounds which may be viewed as potential RCQ molecules, especially if nitrogen atoms are used in the segment that links the quinolines. The approved drug, piperaquine makes an interesting case. Fig. (**4**) shows the essential elements of the RA moiety superimposed on both imipramine and one end of a piperaquine molecule. In fact, one can almost think of CQ itself as a RA pharmacophore, in that the molecule can roughly fit the essential structural elements. Also, binding to PfCRT, a requisite for transport is a trait shared by RA molecules. In fact, PfCRT (mutated or not) may be able to transport some or all of the RA molecules, to various extents [49].

## NEXT-GENERATION RCQ MOLECULES

Future efforts in designing RCQ molecules will be aimed in two directions. First, we are attempting to vary the RA end to a greater extent, with the idea that it may not be necessary to retain strong binding to PfCRT. The other direction will be to vary the substituent pattern on the quinoline ring system, as has been done for CQ-like structures by others, e.g., [50]. Both of these directions may help to reduce the hERG binding which gives rise to the potential cardiotoxicity common to quinoline-based malaria drugs [51]. We already have some evidence that the effects of changing the substituents are not the same as found in CQ and its derivatives.

## THE ROLE FOR RCQ MOLECULES IN THE WORLD-WIDE MALARIA STRATEGY

One could ask why would we wish to develop new 4-aminoquinoline drugs against malaria, especially in the face of the failure of CQ? The first reason is that the drugs are generally inexpensive to produce, particularly on a large scale. The starting materials are not exotic, and the chemistry required to assemble the molecules is quite safe and simple [13-15]. Second, the RCQ molecules are a fairly strong departure from the side chain of CQ, so that there may be minimal cross-resistance to other strains. In fact, we have evaluated several “field-isolated” strain clones developed from infected patients, and found no significant resistance to any of several of the RCQ drugs. Third, the design is exceedingly flexible. This is important because if resistance does emerge against one drug, then it is possible that a next-generation RCQ could take its place. Forth, the physical-chemical properties can be chosen such that its pharmacokinetic parameters would be a good match for another drug in combination therapy, and such ability could be very useful for optimizing drug combinations. Finally, CQ was really a very good drug for nearly half a century. If we are successful in developing RCQ drugs that are even better in terms of efficacy and safety than CQ, then we could use the advantages of quinoline antimalarial drugs in another attempt at eradicating the disease. Given the RCQ potencies, it is conceivable to aim for single-dose radical cures that are hoped for in an elimination / eradication global strategy [52, 53], especially as a part of combination drugs in which the partners are carefully chosen to complement each other’s abilities and properties.

## Figures and Tables

**Fig. (1):**
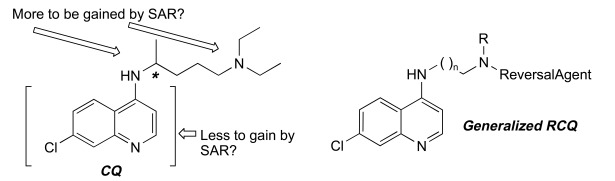
**The original concept leading to RCQ drugs.** Note the chiral center in CQ that we eliminate in a RCQ drug.

**Fig. (2):**
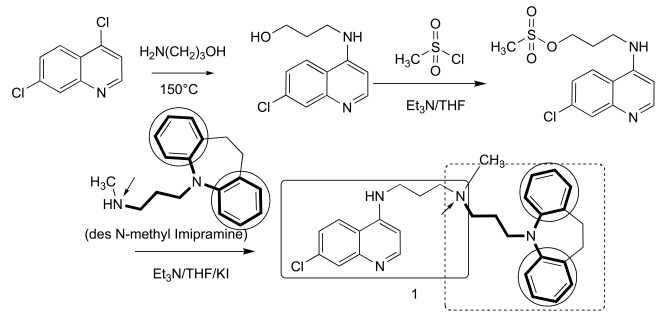
**The synthesis and structure of prototype RCQ molecule, 1** [13]. The hydrogen-bond accepting nitrogen is indicated with an arrow, and the reversal agent aromatic groups are circled; the RA moiety will be shown in bold bonds throughout. In the structure of **1**, the quinoline and linker are contained in the line-box, and the RA moiety is in the dotted box.

**Fig. (3):**
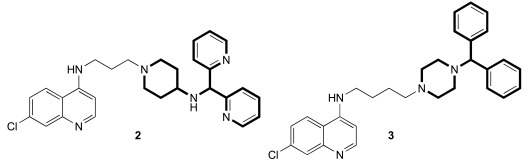
**Two additional key RCQ molecules** [15]. RCQ drug **2** has good water solubility and oral efficacy, and RCQ **3** has the same linker length between the 4-amino group and the next nitrogen as does CQ. Both have high potencies against CQ-sensitive and CQ-resistant *P. falciparum* strains.

**Fig. (4):**
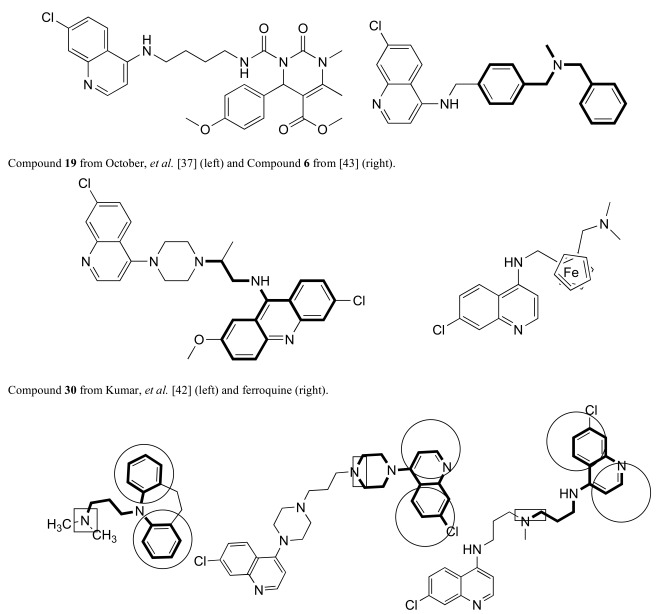
Examples of other molecules may be viewed as sharing aspects of RCQ drugs.

**Table 1.** Selected RCQ Activities against *P. falciparum* [14]

Compound		ClogP *a*	IC50 *b**(*n*M)*		IC_50_ Ratio Dd2/D6	Cytotoxicity *c*(n*M*)
			D6 (CQ^S^)	Dd2 (CQ^R^)		
CQ	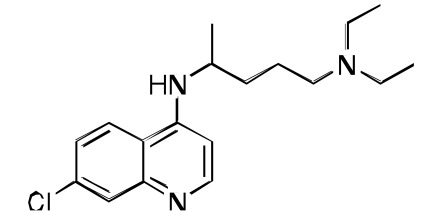	5.1	7	102	15	12000
1	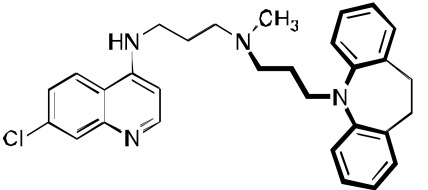	8.9	3	5	1.7	700
2	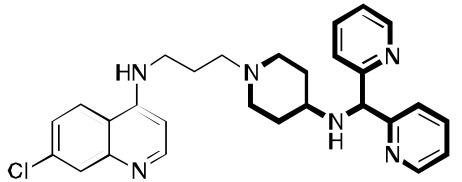	3.5	1.6	1.8	1.1	6500
3	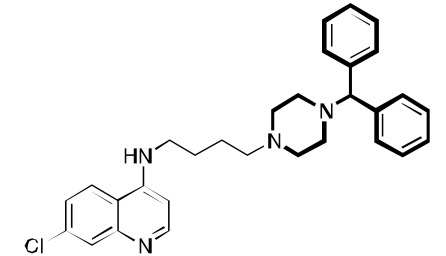	7.5	1.3	1.3	1.0	1100
4	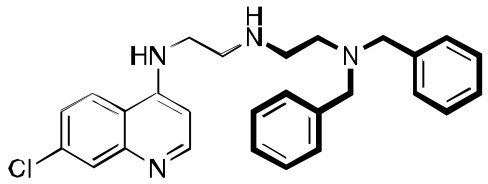	7.0	10	16	1.6	2200
5	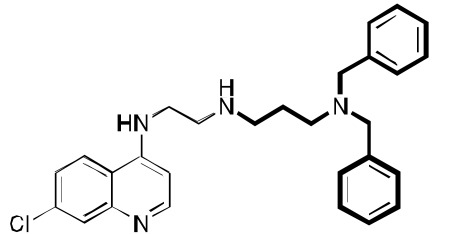	7.3	7	16	2.3	N.D.
6	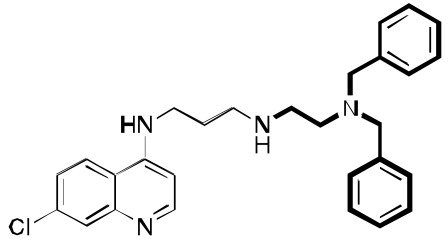	7.3	2	6	3.0	N.D.
7	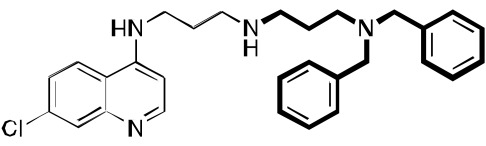	7.6	23	34	1.5	700
8	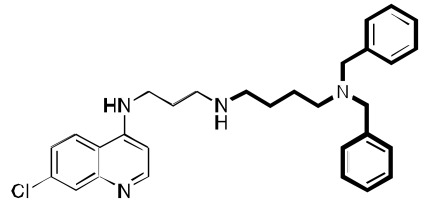	7.4	8	11	1.4	1300

a Evaluated using ChemDraw software.

b Averages of at least 3 runs (± 15%). The uncertainties are estimated based on weighing uncertainties for the various compounds (which are free-bases and often oils), as well as on variability between determinations that were performed on different weeks.

c Cytotoxicities are against mouse spleen lymphocytes. These values are estimated to be ±50%, based on weighing uncertainties for the various compounds (which are free-bases and often oils), as well as on variability between determinations that were performed on different weeks. N.D.: not determined.

## ABBREVIATIONS

CQ: = Chloroquine
PfCRT: = *Plasmodium falciparum* chloroquine resistance transporter
DV: = Digestive vacuole
CQ-resistant: = Chloroquine resistant
CQ-senstive: = Chloroquine sensitive
RA: = Reversal agent
RCQ: = Reversed chloroquine compound
SAR: = Structure-activity relationship
